# Nasal and temporal curvatures of lamina CRIBROSA in myopic eyes

**DOI:** 10.1038/s41598-022-20372-8

**Published:** 2022-10-04

**Authors:** Sooyeon Choe, Yoon Ha Joo, Yong Woo Kim, Young Kook Kim, Jin Wook Jeoung, Jung Chan Lee, Ki Ho Park

**Affiliations:** 1 Department of Ophthalmology, Seoul National University College of Medicine, Seoul, Republic of Korea; 2 Department of Ophthalmology, Seoul National University Hospital, Seoul, Republic of Korea; 3 Interdisciplinary Program in Bioengineering, Seoul National University Graduate School, Seoul, Republic of Korea; 4 Department of Biomedical Engineering, College of Medicine and Institute of Medical and Biological Engineering, Medical Research Center, Seoul National University, Seoul, Republic of Korea; 5 Institute of BioEngineering, Bio-Max Institute, Seoul National University, Seoul, Republic of Korea

**Keywords:** Optic nerve diseases, Medical research

## Abstract

Little is known about the myopic characteristics of lamina cribrosa (LC) curvature. As such, we investigated nasal and temporal LC curvatures in myopia. In this retrospective, cross-sectional study, 144 myopic eyes (refraction < − 2*D*) and 88 non-myopic eyes (refraction > − 0.5*D*) underwent swept-source optical coherence tomography scanning of the LC. The anterior border of LC curvature was delineated with 17 points and interpolated with the “cardinal spline” curve-fitting method. The average curvature indices of the temporal and nasal sides were presented as the temporal and nasal curvatures. Myopic eyes had a mean refraction of − 6.7 ± 2.8*D*, while for non-myopic eyes, the value was 0.3 ± 1.0*D*. Nasal LC curvature was visible in 54 myopia (37.5%) and 42 non-myopia (47.7%) cases (*P* = 0.126), and temporal LC curvature was visible in 142 myopia (98.6%) and 68 non-myopia (77.3%) cases (*P* = 0.001). The nasal LC curvature was significantly larger in myopia than in non-myopia (*P* < 0.001). Contrastingly, the temporal LC curvature was significantly smaller in myopia than in non-myopia (*P* < 0.001). Axial length was associated with larger nasal LC curvature, smaller temporal LC curvature, and larger nasal–temporal LC curvature difference (all *P*’s < 0.05). In myopic relative to non-myopic eyes, LC curvature was decreased temporally and increased nasally.

## Introduction

Myopia is becoming a more important issue in direct proportion to its dramatically increasing prevalence.^1,2^ In myopic eyes, optic discs develop characteristic features. A tilted appearance of the optic disc and temporal crescent, which is now well known as parapapillary atrophy, was the first known feature, as identified by funduscopic examination and optic disc photography.^3–5^ Currently, myopic optic nerve head (ONH) features are being further studied by optical coherence tomography (OCT). Kim et al.^6^ demonstrated that as axial elongation procedes, the temporal border tissue changes to externally oblique, the Bruch’s membrane opening (BMO) enlarges, and the nasal minimum rim width (MRW) elevates.

**Figure 1:**
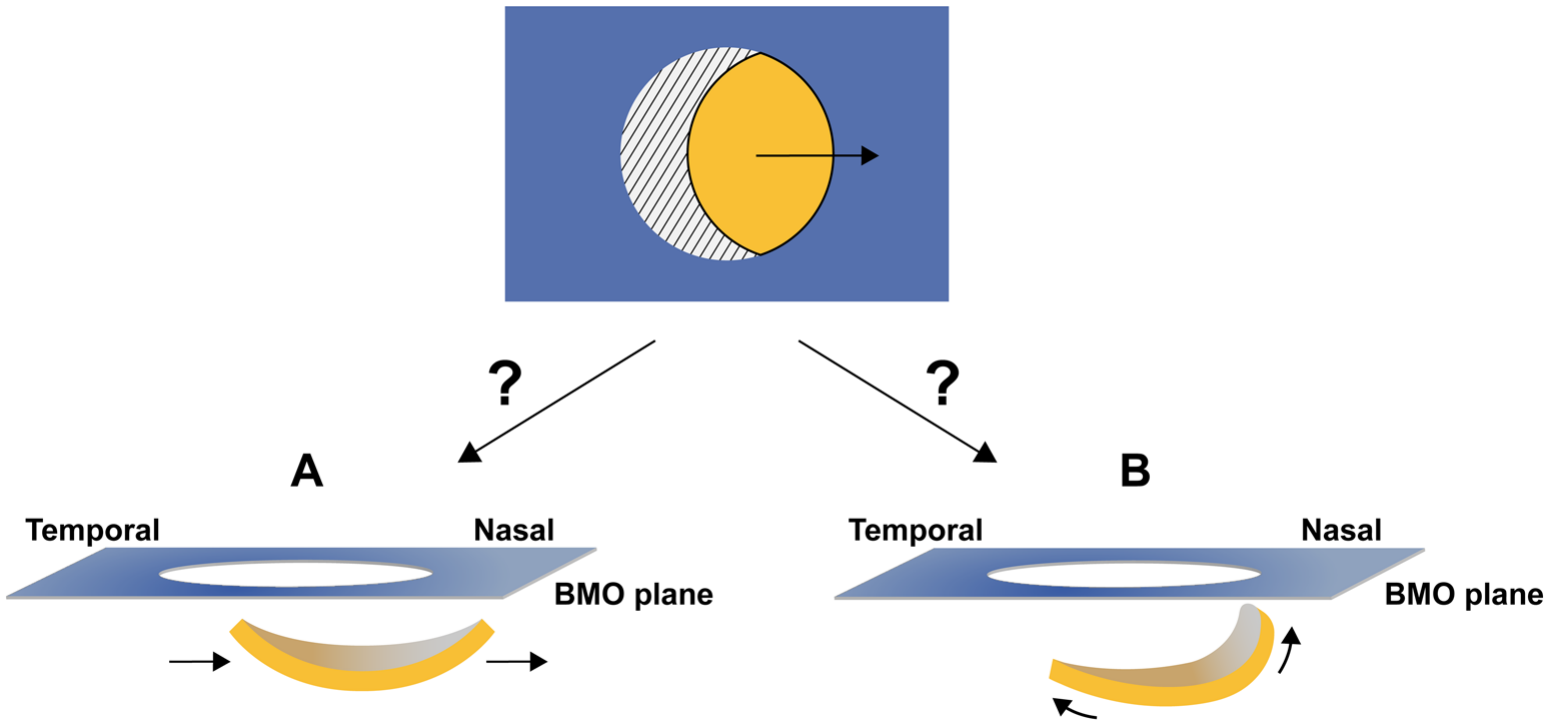
Schematic illustration of hypothetical three-dimensional view of LC in myopic optic nerve head. The above image depicts myopic optic disc, well-known as ‘tilted disc,’ in a two-dimensional view. This ‘two-dimensional tilted disc’ may be, in the three-dimensional view, either (**A**) tilt-like disc appearance or (**B**) real disc tilt.

Some efforts to explain myopic change to the deep structure of the ONH (i.e., lamina cribrosa [LC]), have been made. Lee et al.^7^ emphasized nasal shifting of LC indicated by nasal dragging of the central retinal vascular trunk as a consequence of axial elongation. However, their report could not explain the well-known concept of ‘disc tilt’. At this point the most pertinent question is whether, in myopic eyes, there is real disc tilt or just a tilt-like disc appearance due to nasal shifting of the LC (Fig. 1). To the best of our knowledge, the myopic characteristics of LC curvature, especially including the nasal part of the LC, have yet to be fully elucidated.

In the present study, we aimed to evaluate nasal and temporal curvatures of the LC in myopic eyes and to compare them with those in non-myopic eyes.

## Results

### Subject demographics

Initially, 180 candidate subjects were enrolled. Among them, 64 subjects were excluded due to increased intraocular pressure (IOP), existence of cataract with best-corrected visual acuity (BCVA) less than 20/40, history of ocular surgeries, comorbid retinal diseases, or poor OCT scan quality. Thus, a total of 116 subjects (232 eyes) were involved in this study. All of the subjects were Korean by ethnicity.

The patients were classified into two groups based on refraction: myopia (n = 144; refraction < − 2 *D*) and non-myopia (n = 88; refraction > − 0.5 *D*). The refraction of the myopia group was − 6.7 ± 2.8 *D*, while that of the non-myopia group was 0.3 ± 1.0 *D* (*P* < 0.001). The myopia group was of significantly younger age (52.2 ± 6.7 vs. 60.3 ± 9.5 years; *P* < 0.001), had a smaller proportion of females (50.0 vs. 65.9%; *P* = 0.018), longer axial length (26.1 ± 1.1 vs. 23.6 ± 0.8; *P* < 0.001), and lesser circumpapillary retinal nerve fiber layer (cpRNFL) thickness (71.4 ± 14.4 vs. 82.6 ± 19.5; *P* < 0.001). There were no significant intergroup differences in sex, laterality of eye, BCVA (in logMAR), disc area, cup area or percentage of glaucoma (all *P* > 0.05). The nasal side of the LC was visible in 54 myopic eyes (37.5%) and 42 non-myopic eyes (47.7%) (*P* = 0.126), while the temporal side of the LC was visible in 142 myopic eyes (98.6%) and 68 non-myopic eyes (77.3%) (*P* = 0.001) (Table 1).

**Table 1:** Baseline demographics and descriptive characteristics.

	Myopia (n = 144)	Non-myopia (n = 88)	*P* value
Age (yrs)	52.2 ± 6.7	60.3 ± 9.5	< 0.001*
Female, n (%)	72 (50.0)	58 (65.9)	**0.018**^**†**^
Right eye, n (%)	72 (50.0)	44 (50.0)	1.000^†^
BCVA (logMAR)	0.02 ± 0.1	0.03 ± 0.1	0.345*
Refraction (Diopter)	− 6.7 ± 2.8	0.3 ± 1.0	< 0.001*
Axial length (mm)	26.1 ± 1.1	23.6 ± 0.8	< 0.001*
IOP (mmHg)	13.0 ± 2.7	13.3 ± 2.4	0.421*
Average cpRNFL thickness (μm)	71.4 ± 14.4	82.6 ± 19.5	< 0.001*
MD (dB)	− 5.7 ± 6.4	− 4.7 ± 6.4	0.284*
Disc area (mm^2^)^‡^	2.0 ± 0.7	1.9 ± 0.7	0.496*
Cup area (mm^2^)^‡^	1.3 ± 0.8	1.3 ± 0.7	0.905*
Glaucoma, n (%)	100 (69.9)	59 (68.6)	0.835^†^
Nasal LC visibility, n (%)	54 (37.5)	42 (47.7)	0.126^§^
Temporal LC visibility, n (%)	142 (98.6)	68 (77.3)	**0.001**^§^

Except where indicated otherwise, values are mean ± standard deviation or number (%).

*Comparison was performed using the Student *t* test.

^†^Comparison was performed using the two proportion z-test.

^‡^Corrected area calculated in consideration of the magnification factors related to the SD-OCT camera and the eye by substituting the subject’s axial length.

^§^Comparison was performed using the chi-squared test.

Statistically significant values are shown in bold (*P* < 0.05).

BCVA: best-corrected visual acuity; cpRNFL: circumpapillary retinal nerve fiber layer; IOP: intraocular pressure; LC: lamina cribrosa; MD: mean deviation.

The baseline demographics and descriptive characteristics of the eyes with visible nasal and temporal LC are shown in Table 2. If both eyes of a candidate were eligible, the eye without glaucoma (or less severe glaucoma with a higher mean deviation [MD] value) was selected for the subsequent analysis. Forty-five myopic eyes of 45 subjects and 30 non-myopic eyes of 30 subjects were included for the subsequent analysis. The refraction of the myopia group was − 6.6 ± 2.6 D, while that of the non-myopia group was 0.5 ± 1.1 D (*P* < 0.001). The myopia group was of younger age (52.3 ± 7.2 vs. 60.5 ± 10.4), and had longer axial length (26.3 ± 1.2 vs. 23.5 vs. 0.8) and thinner cpRNFL thickness (71.7 ± 13.6 vs. 86.8 ± 20.5) (all *P*’s < 0.001).

**Table 2:** Baseline demographics and descriptive characteristics with visible nasal LC.

	Myopia (n = 45)	Non-myopia (n = 30)	*P* value
Age (yrs)	52.3 ± 7.2	60.5 ± 10.4	< 0.001*
Female, n (%)	25 (55.6)	22 (73.3)	0.121^†^
Right eye, n (%)	15 (33.3)	11 (36.7)	0.764^†^
BCVA (logMAR)	0.007 ± 0.1	0.048 ± 0.1	0.056*
Refraction (Diopter)	− 6.6 ± 2.6	0.5 ± 1.1	< 0.001*
Axial length (mm)	26.3 ± 1.2	23.5 ± 0.8	< 0.001*
IOP (mmHg)	13.0 ± 2.1	13.5 ± 2.5	0.351*
Average cpRNFL thickness (μm)	71.7 ± 13.6	86.8 ± 20.5	< 0.001*
MD (dB)	− 5.0 ± 6.3	− 4.3 ± 5.9	0.645*
Disc area (mm^2^)^‡^	2.0 ± 0.5	1.8 ± 0.4	0.076*
Cup area (mm^2^)^‡^	1.1 ± 0.5	1.0 ± 0.5	0.514*
Glaucoma, n (%)	32 (71.1)	20 (66.7)	0.689^†^

Except where indicated otherwise, values are mean ± standard deviation or number (%).

*Comparison was performed using the Student *t* test.

^†^Comparison was performed using the two proportion z-test.

^‡^Corrected area calculated in consideration of the magnification factors related to the SD-OCT camera and the eye by substituting the subject’s axial length.

Statistically significant values are shown in bold (*P* < 0.05).

BCVA: best-corrected visual acuity; cpRNFL: circumpapillary retinal nerve fiber layer; IOP: intraocular pressure; LC: lamina cribrosa; MD: mean deviation.

### Comparison of LC curvature

The nasal and temporal curvatures of the LC were compared between the myopic and non-myopic eyes with visible LC. Nasal LC curvature in myopic eyes (2.3 ± 0.5 [1/mm]) was significantly larger than that in-non myopic eyes (1.8 ± 0.3 [1/mm]) (*P* < 0.001). Meanwhile, temporal LC curvature in myopic eyes (0.9 ± 0.7 [1/mm]) was smaller than that in non-myopic eyes (1.7 ± 0.6 [1/mm]), with statistical significance (*P* < 0.001). The difference between nasal–temporal curvature was significantly larger in myopic eyes than in non-myopic eyes (*P* < 0.001) (Table 3).

**Table 3:** Comparison of nasal and temporal curvatures of LC between myopia and non-myopia.

	Myopia with visible nasal LC (n = 45)	Non-myopia with visible nasal LC (n = 30)	*P* value
Nasal LC curvature (1/mm)	2.3 ± 0.5	1.8 ± 0.3	< 0.001*
Temporal LC curvature (1/mm)	0.9 ± 0.7	1.7 ± 0.6	** < 0.001**^†^
LC curvature difference (nasal–temporal) (1/mm)	1.4 ± 0.9	0.1 ± 0.8	< 0.001*

Except where indicated otherwise, values are mean ± standard deviation.

*Comparison was performed using the Student t test.

^†^Comparison was performed using the Wilcoxon signed-rank test.

Statistically significant values are shown in bold (*P* < 0.05).

LC: lamina cribrosa.

### Factors associated with LC curvature

The factors associated with the LC curvature parameters were analyzed using a multivariate linear regression model. Multicollinearity was checked, and all of the variance inflation factors (VIFs) were less than 2.5. Axial length was significantly associated with larger nasal LC curvature (*β* = 0.128, *P* = 0.011), smaller temporal LC curvature (*β* = − 0.278, *P* < 0.001) and larger nasal–temporal LC curvature difference (*β* = 0.406, *P* < 0.001). Smaller disc area was associated with larger nasal LC curvature (*β* = − 0.034, *P* = 0.021) and larger nasal–temporal LC curvature difference (*β* = − 0.648, *P* = 0.027) (Table 4).

**Table 4:** Factors associated with LC curvature parameters.

	*ß*	Standard Error	95% CI	*P* value*
**Nasal LC curvature (1/mm)**				
Age (yrs)	− 0.013	0.008	− 0.029 to 0.003	0.110
Sex (female)	− 0.244	0.145	− 0.537 to 0.049	0.101
Axial length (mm)	0.128	0.048	0.031 to 0.226	**0.011**
IOP (mmHg)	− 0.012	0.027	− 0.067 to 0.042	0.651
Disc area (mm^2^)^†^	− 0.034	0.161	− 0.708 to − 0.060	**0.021**
Average cpRNFLT (μm)	0.004	0.004	− 0.004 to 0.013	0.370
MD (dB)	− 0.010	0.011	− 0.032 to 0.012	0.358
Glaucoma	0.002	0.162	− 0.325 to 0.329	0.989
**Temporal LC curvature (1/mm)**				
Age (yrs)	0.005	0.010	− 0.016 to 0.026	0.650
Sex (female)	0.174	0.187	− 0.203 to 0.551	0.357
Axial length (mm)	− 0.278	0.062	− 0.403 to − 0.153	** < 0.001**
IOP (mmHg)	− 0.007	0.035	− 0.078 to 0.063	0.832
Disc area (mm^2^)^†^	0.264	0.207	− 0.153 to 0.681	0.208
Average cpRNFLT (μm)	− 0.005	0.006	− 0.016 to 0.007	0.404
MD (dB)	− 0.00002	0.014	− 0.029 to 0.029	0.999
Glaucoma	0.114	0.208	− 0.306 to 0.535	0.587
**LC curvature difference (nasal–temporal) (1/mm)**				
Age (yrs)	− 0.018	0.014	− 0.047 to 0.011	0.214
Sex (female)	− 0.418	0.256	− 0.935 to 0.099	0.110
Axial length (mm)	0.406	0.085	0.235 to 0.577	** < 0.001**
IOP (mmHg)	− 0.005	0.048	− 0.101 to 0.092	0.919
Disc area (mm^2^)^†^	− 0.648	0.283	− 1.219 to − 0.077	**0.027**
Average cpRNFLT (μm)	0.009	0.008	− 0.007 to 0.025	0.265
MD (dB)	− 0.010	0.020	− 0.050 to 0.029	0.601
Glaucoma	− 0.112	0.285	− 0.688 to 0.464	0.697

*Statistical analysis was performed using multivariate linear regression model.

^†^Corrected area calculated in consideration of the magnification factors related to the SD-OCT camera and the eye by substituting the subject’s axial length.

Statistically significant values are shown in bold (*P* < 0.05).

### Representative cases

**Figure 2:**
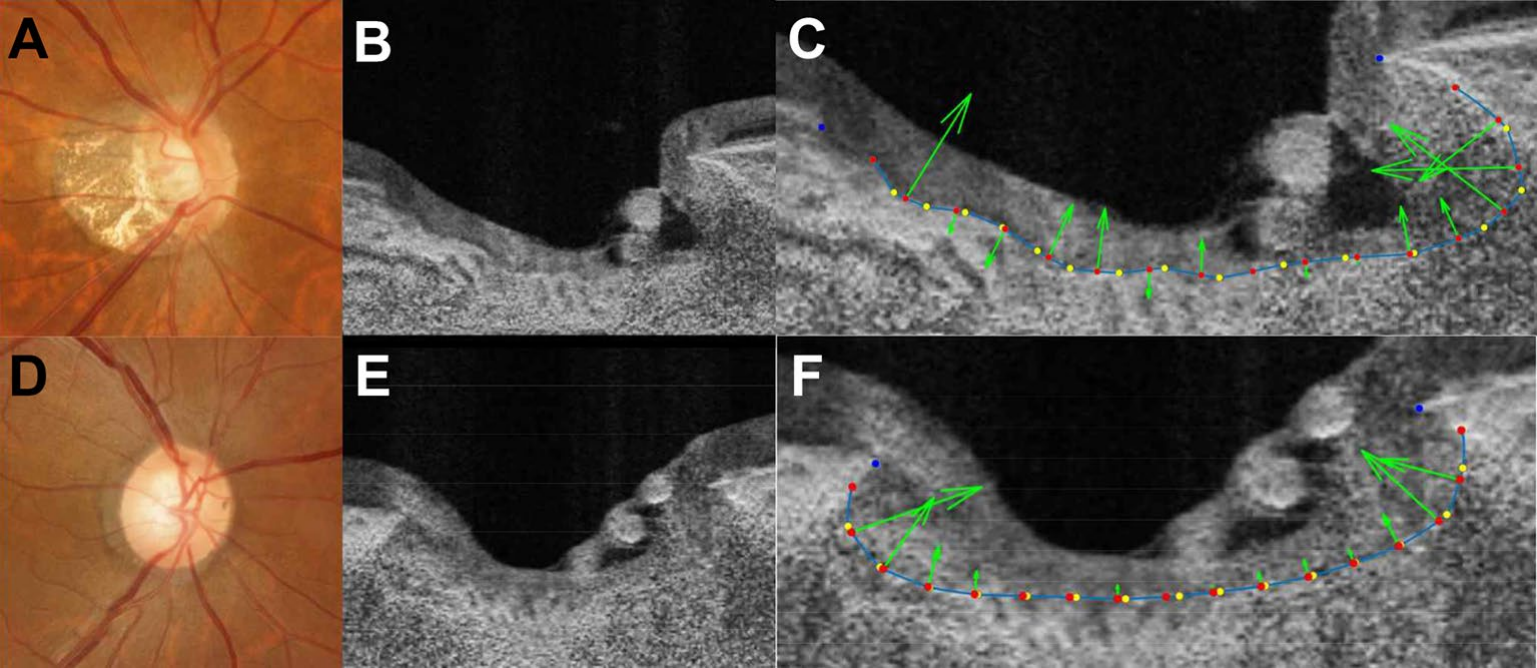
Representative cases with visible nasal LC. Representative images of myopic eyes (**A–C**) and non-myopic eyes (**D–F**) are presented. The images of myopic eye are from a 51-year-old female with refraction of − 7.0 *D* and an axial length of 26.1 mm. The images of non-myopic eye are from a 66-year-old female with refraction of + 2.6 *D* and an axial length of 23.9 mm. The first column (**A,D**) shows disc photographs. The second column (**B,E**) provides horizontal SS-OCT optic disc B-scans. The third column (**C,F**) provides the calculated vectors of LC curvature (green arrows), as measured from each equally-divided dot (red dots). The nasal LC curvature was 1.80 (1/mm) and the temporal LC curvature was 0.78 (1/mm) for the myopic eye and 1.56 (1/mm) and 1.75 (1/mm), respectively for the non-myopic eye.

Figure 2 shows representative cases of myopic and non-myopic eyes with visible nasal LC. In the first row are the findings for the eye of a 51-year-old myopic female. The axial length was 26.1 mm and the refraction was − 7.0 *D*. The nasal LC curvature was 1.80 (1/mm) and the temporal LC curvature was 0.78 (1/mm). In the second row are the results for the eye of a 66-year-old non-myopic female. The axial length was 23.9 mm and the refraction was + 2.6 *D*. The nasal LC curvature was 1.56 (1/mm) and the temporal LC curvature was 1.75 (1/mm).

## Discussion

This study investigated the nasal and temporal LC curvatures of myopic and non-myopic eyes. We found that the nasal side of LC curvature is larger and the temporal side is smaller in myopia relative to non-myopia. Additionally, we evaluated the factors associated with nasal and temporal LC curvatures.

**Figure 3:**
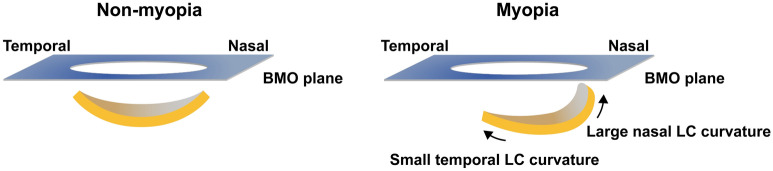
Schematic illustration showing nasal and temporal LC curvature in non-myopic and myopic eyes. Unlike non-myopic eyes, in myopic eyes, the nasal LC curvature shows an abruptly tilted appearance while the temporal LC curvature shows a flattened appearance.

The association of myopia with morphologic ONH change (i.e., of the LC) has been investigated widely. Lee et al.^7^ found that during myopic progression, the position of the central retinal vascular trunk, which is embedded in the LC, was dragged nasally. Another study^8^ from the same group demonstrated that during axial elongation, the temporal border tissue length stretches to the nasal side while its angle changes from internally oblique to externally oblique. Kim et al.^6^ previously discovered that with elongation of axial-length, nasal MRW increases, which suggests that nasal peripapillary tissue elevation occurs with progression of myopia. Our findings can supplement results from previous studies on myopic ONH change, especially with regard to three-dimensional change of LC curvature. We can postulate that as axial elongation proceeds during myopic change, the central part of the LC moves nasally and causes the nasal part to tilt abruptly and curve. At the same time, the temporal LC would be stretched, thereby coming to take on a more flattened appearance (Fig. 3).

The LC plays a primary role in the pathogenesis of glaucoma^9–14^. Deformation of the LC causes retinal ganglion cell (RGC) axonal injury, which leads in turn to glaucoma. LC deformation contributory to glaucoma has manifested mainly as localized laminar defects^15–19^ and LC thickness^20,21^ or depth change^11,22–26^. The findings of the present study possibly explain the phenomenon of topographic distribution of localized LC defect and its association with glaucoma. Previous studies have shown that large LC pores and LC defects are located mostly on the temporal rather than the nasal side^27,28^. We can postulate that axial-elongation-associated stretching of the temporal LC generates tensile force that results in localized LC defect on the temporal side. Also, compromised LC structure can lead to vulnerability of RGC axons. On the other hand, it may also be postulated that as the nasal part of the LC tilts, compression of LC structures therein is incurred, and optic nerves running through nasal LC pores might be injured, thus generating temporal visual-field defects. This explanation supports a previous report^29^ that a temporal visual-field defect group with myopia showed a higher optic disc tilt ratio than did a typical glaucomatous-field defect group.

In regard to morphologic deformation of the LC, previous studies have focused mainly on LC depth and thinning to explain the association with glaucoma^20,21,26,30,31^. However, in order to understand this association, three-dimensional deformation of the LC in the myopic ONH should be investigated. Our study is novel in having evaluated nasal LC curvatures where visualization of the LC is relatively difficult on OCT images due to shadowing from the nasal neuroretinal rim. As such, it can further elucidate previous assumptions regarding axial-elongation-associated ONH changes and myopic optic neuropathies such as myopic glaucoma.

Our study has several limitations. First, although swept-source OCT (SS-OCT) is comparable to enhanced depth imaging OCT in LC imaging^32^, it sometimes has a limitation in visualizing deep ONH structures that include the LC. To reduce selection bias, we excluded cataract eyes with vision worse than 20/40, ocular surgery history and/or poor-quality OCT images. Also, to overcome the ambiguity inherent in delineating the anterior border of the LC, we performed a reliability test to assess inter-rater difference (Kappa = 0.90; almost perfect). Second, both patients with and without glaucoma were included in this study. To minimize the effect of glaucoma and IOP on LC curvature, we excluded patients with IOP over the normal range (> 21 mmHg) and performed a multivariate linear regression analysis for IOP, cpRNFL thickness, MD and presence of glaucoma as covariates. Third, all of the subjects were Korean by ethnicity. Since optic disc head properties manifest racial differences^33,34^, our results cannot be generalized to all ethnicities. Fourth, the cross-sectional nature of this study implies that it can show only the association of myopia with features of LC curvature. Further longitudinal cohort study should be performed to more fully elucidate the causal relationship between myopia and LC curvature and its effect on glaucoma. Lastly, the imbalances of age, size, and percentage of LC visibility between the myopic and non-myopic groups may have caused selection bias. One therefore should be cautious in interpreting the results; in fact, further study with a larger cohort will be needed to minimize bias. Also, multiple factors should be sought out and grouped by PCA plot when investigating the factors associated with LC curvature.

In conclusion, myopic eyes show characteristic features of LC curvature. The nasal LC bends more and the temporal LC flattens as axial length elongates. This finding can help to broaden understanding of myopic LC configurations and the pathophysiology of myopic optic neuropathy.

## Methods

This was a single-center retrospective study performed at Seoul National University Hospital. The study was approved by the Institutional Review Board of Seoul National University Hospital (IRB number: 2202-037-1298), which adhered to the tenets of the Declaration of Helsinki. Written informed consent was waived due to the retrospective nature of the study by the Institutional Review Board of Seoul National University Hospital.

### Study subjects

All of the subjects had visited the Seoul National University Hospital Glaucoma Clinic in Seoul, Korea, between January 2015 and July 2022. We reviewed medical records retrospectively, and included eligible adult (over 40 years old) subjects. Based on refraction, the patients were classified into two groups: myopia (refraction < − 2 *D*) and non-myopia (refraction > − 0.5 *D*).

Patients were excluded from further analysis for any of the following reasons: (1) IOP over 21 mmHg by Goldmann applanation tonometry, (2) existence of cataract with BCVA worse than 20/40 in Snellen equivalent, (3) history of intraocular surgery or trauma, (4), any evidence of pathologic myopia or high-myopia-associated optic neuropathy, or (5) poor optical coherence tomography scan quality. Patients for whom both eyes qualified for inclusion were enrolled.

All of the enrolled patients underwent a complete ophthalmic examination, including BCVA assessment, refraction by keratorefractometry (KR-800A, Topcon, Tokyo, Japan), axial-length measurement (Axis II PR; Quantel Medical, Inc., Bozeman, MT, USA), slit-lamp biomicroscopy, Goldmann applanation tonometry (Haag-Streit, Koniz, Switzerland), dilated fundus examination, stereo optic disc photography, red-free retinal nerve fiber layer photography (TRC-50IX; Topcon Corporation, Tokyo, Japan), and 24-2 SITA Standard protocols of Humphrey VF (Analyzer II; Carl Zeiss Meditec, Dublin, CA, USA). Additionally, Cirrus HD-OCT (Carl Zeiss Meditec) and SS-OCT (Deep Range Imaging [DRI] OCT, Triton, Topcon) were performed at the same visit. The disc area and cup area were obtained from Cirrus OCT. The corrected disc and cup areas were calculated, in consideration of OCT-camera- and human-eye-related magnification factors, with the following equation, which calculation process is described in detail in previous reports^35–37.^

$$\begin{aligned} {\text{Corrected area }}\left( {{\text{mm}}^{{2}} } \right) & = {3}.{382}^{{2}} \times 0.0{13}0{6}^{{2}} \times \left( {\text{axial length} {-} {1}.{82}} \right)^{{2}} \\ & \quad \times \left( {{\text{OCT}}-{\text{computed area}}} \right) \\ \end{aligned}$$

### SS-OCT imaging

SS-OCT imaging was performed after dilation of the pupil. Three-dimensional disc scans, covering 6 mm × 6 mm area and comprising 512 A-scans × 256 B-scans, were obtained. The protocol captures color fundus photos as well as optic disc scans, allowing for simultaneous evaluation between OCT B-scans and color optic disc photos with the built-in review program (IMAGEnet 6 Version 1.25, Topcon). The cpRNFL thickness was automatically measured with the built-in review program (IMAGEnet 6 Version 1.25, Topcon). The obtained OCT scans were reviewed, and those of poor quality, that included, for example, motion artifacts, defocusing and/or poor centration, were excluded from further analysis.

### Evaluation of LC curvature

**Figure 4:**
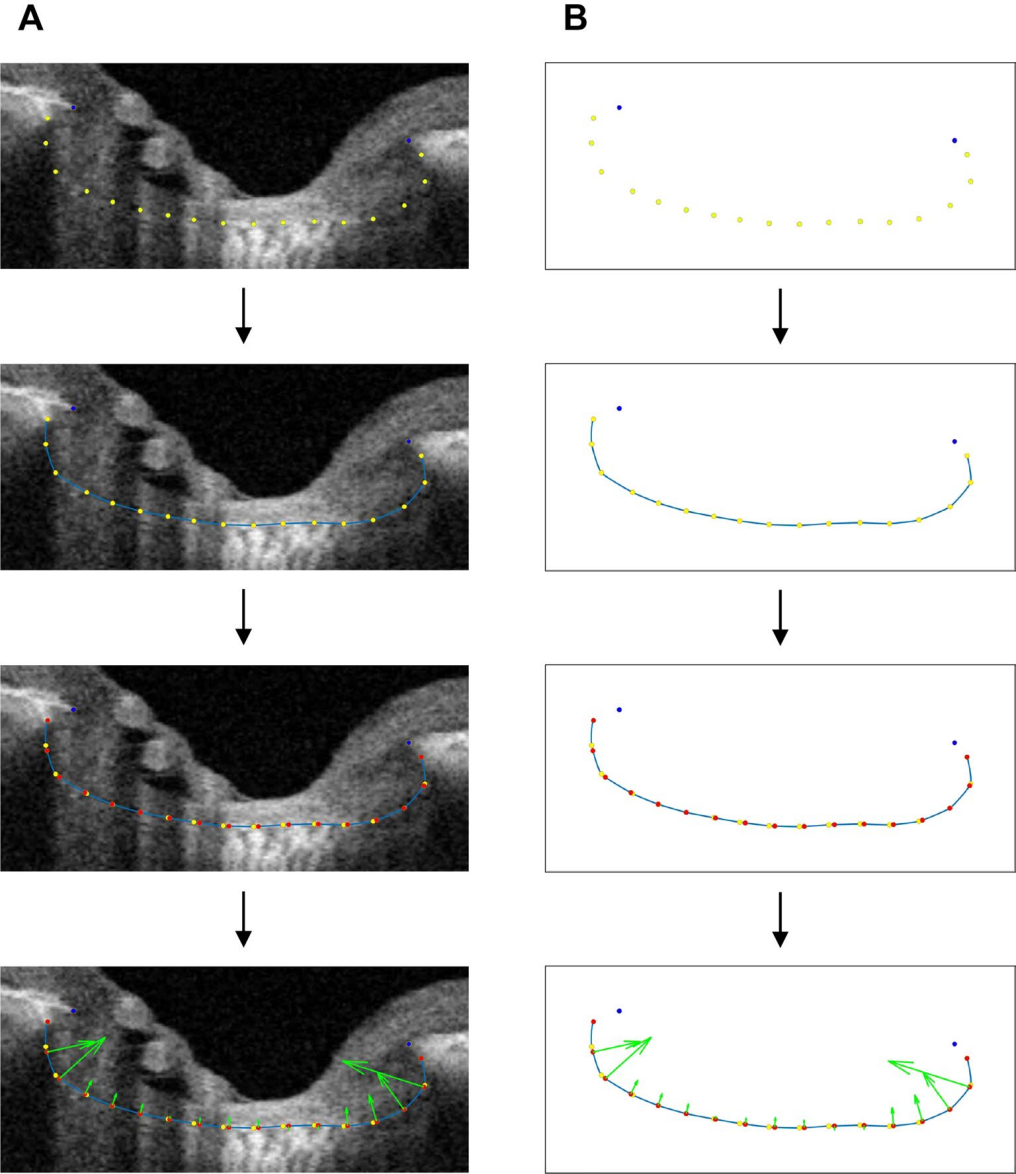
Diagram describing methodology of LC curvature measurement. **(A)** BMO points (blue dots) with 17 yellow dots delineating the anterior surface of the LC are shown. The yellow dots were interpolated with the cardinal spline curve-fitting method (blue curve). The blue curve was separated into 17 points at regular intervals (red points). The circumcenter of the triplet of adjacent red points along the blue curve was calculated as the curvature index (green arrow). The average of the nasal side is presented as the nasal LC curvature, and the average of the temporal side is presented as the temporal LC curvature. **(B)** The right column provides a schematic version of each corresponding left-column image.

In the present study, three horizontal B-scans were selected and analyzed from the center (middle one-third), superior (upper one-third), and inferior (lower one-third) horizontal meridians, and then averaged for the analysis. To improve LC visibility, we enhanced the OCT images using the adaptive compensation technique^38–40^. In the enhanced images, the two BMO points were connected to define the BMO reference plane. The middle point of the BMO distance was identified, from which a perpendicular line was drawn. The meeting point of this line and the anterior surface of the LC was marked as the midline reference point. The anterior surface of the LC between the midline reference point and the nasal and temporal end points, respectively, was divided into 8 equivalently spaced points. The points were interpolated with the “cardinal spline” curve-fitting method^41^. To separate the spline into 17 points at regular intervals, arc length parameterization was performed. The circumcenter of the triplet of adjacent points along the curve was calculated as the curvature index. The average curvature indices of the temporal and nasal sides were presented as the temporal and nasal curvatures (Fig. 4). The above-referenced calculations were performed using MATLAB R2021 (MathWorks, Inc., MA, USA). To determine the interobserver measurement reproducibility of LC curvatures, the intraclass correlation coefficient (ICC) with its confidence interval (CI) was calculated by two independent examiners (S.C. and Y.K.K.) in a masked fashion. The strength of agreement was almost perfect (Kappa: 0.90).

### Data analysis

Continuous variables (age, BCVA, refraction, axial length, IOP, cpRNFL thickness, disc area, cup area and LC curvatures) were compared by Student *t* test or by Wilcoxon signed-rank test. Categorical variables (sex, laterality, presence of glaucoma and percentage of LC visibility) were compared using the two proportion z-test or the chi-squared test. A multivariate linear regression model was used to identify factors associated with nasal or temporal LC curvature (scatterplots of variables are provided in Supplementary Information Fig. S1). The statistical analysis was performed by MATLAB R2021 (MathWorks, Inc.) and STATA 13.0 (StataCorp LP, College Station, TX, USA). Except where stated otherwise, the data are presented herein as the mean ± standard deviation, and the level of statistical significance was set at 2-sided *P* < 0.05.

## Supplementary Information

Supplementary Information.

## Data Availability

The datasets analysed during the current study are not publicly available due to patient data privacy policy, but are available from the corresponding author (K.H.P.) on reasonable request.

## Abbreviations

BCVA: Best-corrected visual acuity
BMO: Bruch’s membrane opening
cpRNFL: Circumpapillary retinal nerve fiber layer
IOP: Intraocular pressure
LC: Lamina cribrosa
MD: Mean deviation
MRW: Minimum rim width
OCT: Optical coherence tomography
ONH: Optic nerve head
RGC: Retinal ganglion cell
SS-OCT: Swept-source optical coherence tomography
