# Supplementary material for: Nasal and temporal curvatures of lamina CRIBROSA in myopic eyes
Source: Sci Rep. 2022 Oct 4;12:16561. doi: 10.1038/s41598-022-20372-8 (PMC9532408; doi:10.1038/s41598-022-20372-8)
Supplement: Supplementary file 1 — Supplementary Information. [file 41598_2022_20372_MOESM1_ESM.pdf]

Supplementary Information

Nasal and Temporal Curvatures of Lamina Cribrosa in Myopic Eyes

Sooyeon Choe, MD[1,2], Yoon Ha Joo, PhD[3], Yong Woo Kim, MD, PhD[1,2], Young Kook Kim, MD[1,2], Jin Wook Jeoung, MD, PhD[1,2], Jung Chan Lee[4,5], Ki Ho Park, MD, PhD[1,2*]

[1] Department of Ophthalmology, Seoul National University College of Medicine, Seoul, Korea

[2] Department of Ophthalmology, Seoul National University Hospital, Seoul, Korea

[3] Interdisciplinary Program in Bioengineering, Seoul National University Graduate School, Seoul, Republic of Korea

[4] Department of Biomedical Engineering, College of Medicine and Institute of Medical and Biological Engineering, Medical Research Center, Seoul National University, Seoul, Korea

[5] Institute of BioEngineering, Bio-Max Institute, Seoul National University, Seoul, Korea

[*]Corresponding author's email: kihopark@snu.ac.kr

Supplementary Figure S1. Scatter plots of variables used in multivariate linear regression model

Supplementary Figure S1. Scatter plots of variables used in multivariate linear regression model

(A) Nasal LC curvature

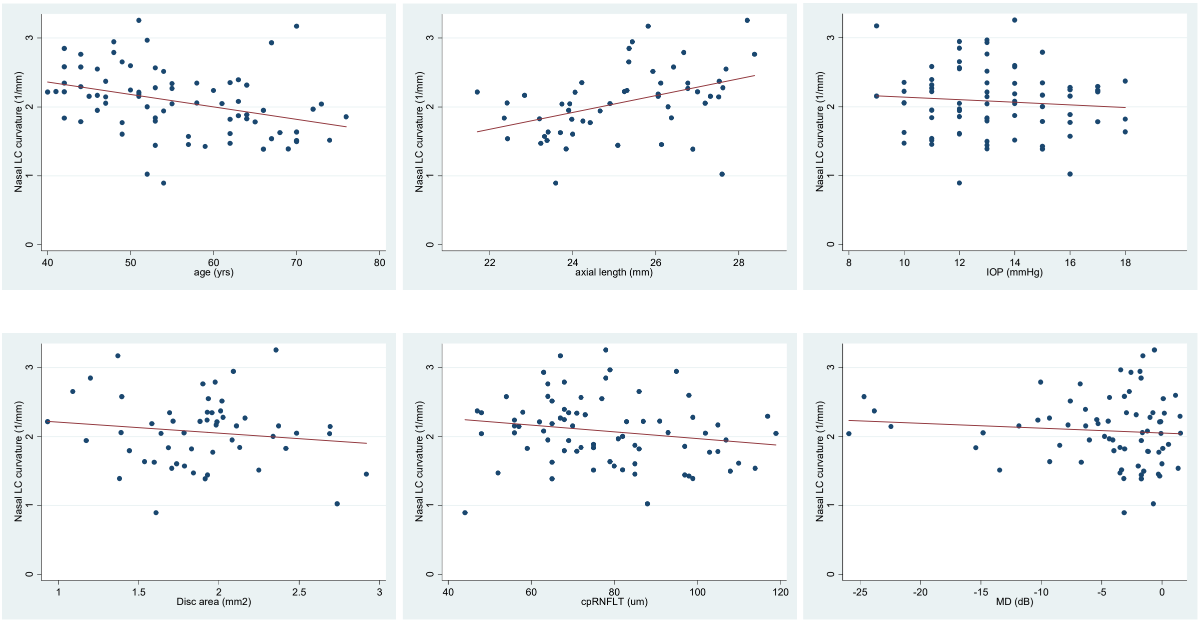

(B) Temporal LC curvature

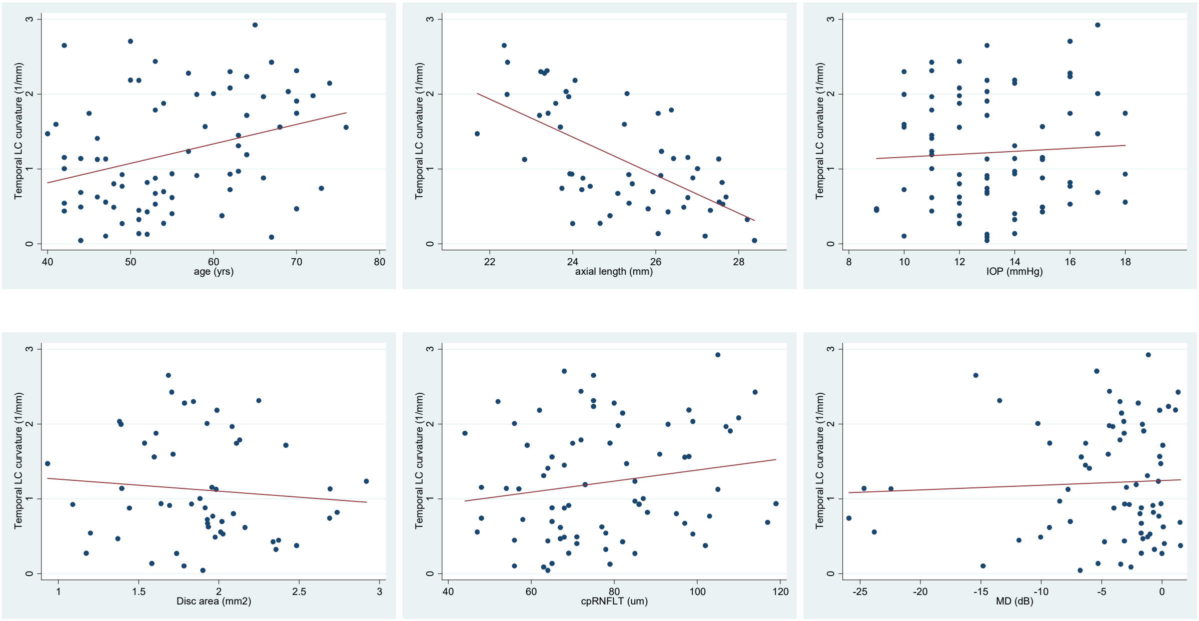

Acronyms: IOP = intraocular pressure; cpRNFLT = circumpapillary retinal nerve fiber layer thickness; MD = mean deivation; LC = lamina cribrosa
